# A Renal Colic Mimic - Wunderlich Syndrome: A Case Report

**DOI:** 10.7759/cureus.11242

**Published:** 2020-10-29

**Authors:** Jaseem Sirajudeen, Nishan K Purayil, Arif Parambath, Muhammed Kayakkool

**Affiliations:** 1 Internal Medicine, Hamad Medical Corporation, Doha, QAT; 2 Radiology, Hamad Medical Corporation, Doha, QAT

**Keywords:** wunderlich syndrome, perinephric hematoma

## Abstract

Wunderlich syndrome is a rare clinical syndrome characterized by the sudden onset of spontaneous, nontraumatic hemorrhage into renal subcapsular and retroperitoneal region. We present the case of a 24-year-old hypertensive who presented with acute flank pain and was found to have perinephric hematoma. He was managed conservatively and the follow-up revealed complete resolution of the hematoma with no structural abnormality of kidney. His connective tissue disorder/vasculitis work up was also normal.

## Introduction

Acute abdominal pain is a common presenting complaint in patients visiting the ED. Such cases often prove a diagnostic challenge to emergency physicians, especially if the patients are hemodynamically unstable, elderly, and women of childbearing age group [1]. Spontaneous renal hemorrhage (Wunderlich syndrome) can mimic such a presentation. Neoplasm is the most common cause; however, various other etiologies have been identified [2]. Patients might even require surgical intervention for control of bleeding.

## Case presentation

A 24-year-old male presented to our ED with complaints of right flank pain, with sudden onset, and rapid progression. The pain was localized to the right flank. There was no associated radiation of pain, fever, hematuria, or history of trauma. He also complained of two episodes of vomiting. He had been on regular medication for hypertension and chronic renal failure.

Upon arrival, the patient was afebrile, blood pressure (BP) was 114/70, pulse rate was 88 beats/min, room air saturation 100%, and respiratory rate was 22 breaths/min. Systemic examination of the abdomen was soft with tenderness over the right lower quadrant, with a palpable mass over the renal angle. Another systemic examination was unremarkable. Blood investigations revealed hemoglobin (Hgb) 10.7 g/dL (baseline Hgb 13.7 g dL), creatinine 222 umol/L, urine microscopy blood 3+, and protein 3+ (Table 1).

**Table 1:** Laboratory values. WBC, white blood cells; Hgb, hemoglobin; eGFR, estimated glomerular filtration rate; bilirubin T, bilirubin total; ALT, alanine transaminase; AST, aspartate amino transferase; TIBC, total iron binding capacity; PTH, parathyroid hormone; POC Urine, point of care urine analysis; LEUKO, leukocytes; NIT, nitrites; PRO, protein; GLU, glucose; KET, ketones; URO, urobilinogen; BIL, bilirubin; SG, specific gravity; BLD, blood

Group	Test	Result	Normal range
General hematology	WBC	6.90 x 10^3/uL	4.00-10.00
	Hgb	13.1 g/dL	13.0-17.0
	Platelet	287 x 10^3/uL	150-400
Blood chemistry	Urea	7.00 mmol/L	2.76-8.07
	Creatinine	242 umol/L	62-106
	eGFR	27 mL/min	
	Sodium	139 mmol/L	136-145
	Potassium	3.4 mmol/L	3.5-5.1
	Chloride	99 mmol/L	98-107
	Bicarbonate	26 mmol/L	22-29
	Calcium	2.26 mmol/L	2.15-2.50
	Calcium Corr	2.34 mmol/L	2.20-2.55
	Phosphorus	1.13 mmol/L	0.81-1.45
	Magnesium	0.82 mmol/L	0.66-1.07
	Bilirubin T	5 umol/L	0-21
	Total protein	69 g/L	66-87
	Albumin Lvl	36.0 g/L	35.0-52.0
	Uric acid	373 umol/L	202-416
	Alk phos	76 U/L	40-129
	ALT	16 U/L	0-41
	AST	17 U/L	0-40
	Cholesterol	4.0 mmol/L	<5.2
	Triglyceride	1.1 mmol/L	<1.7
	Iron	5 umol/L	6-35
	TIBC	53 umol/L	45-80
	Transferrin	2.1 g/L	2.0-3.6
	Fe% saturation	9%	15-45
	Glucose	4.6 mmol/L	3.3-5.5
Endocrinology	Vit D	13 ng/mL	
	PTH	75.6 pg/mL	15.0-65.0
	Vit B12	252.0 pmol/L	145.0-596.0
Autoimmune diseases	Anti cardiolipin Ab IgG	1.00 GPL	
	Anti cardiolipin Ab IgM	<0.80 MPL	
	Anti GBM Ab	<1.9 U/mL	
	Anti ds DNA	0.0.5 IU/L	
	ANCA	Negative	
	C3	137 mg/dL	90-180
	C4	27 mg/dL	10-40
Bacteriology	Blood culture	No growth	
POC urine	Ur pH - POC	6.0	
	Ur LEUKO - POC	Negative	
	Ur NIT - POC	Negative	
	Ur PRO - POC	3+	
	Ur GLU - POC	Negative	
	Ur KET -POC	Negative	
	Ur URO - POC	3.2 umol/L	
	Ur BIL - POC	Negative	
	Ur SG - POC	1.020	
	Ur BLD - POC	3+	
Urine culture	No growth		

**Figure 1:**
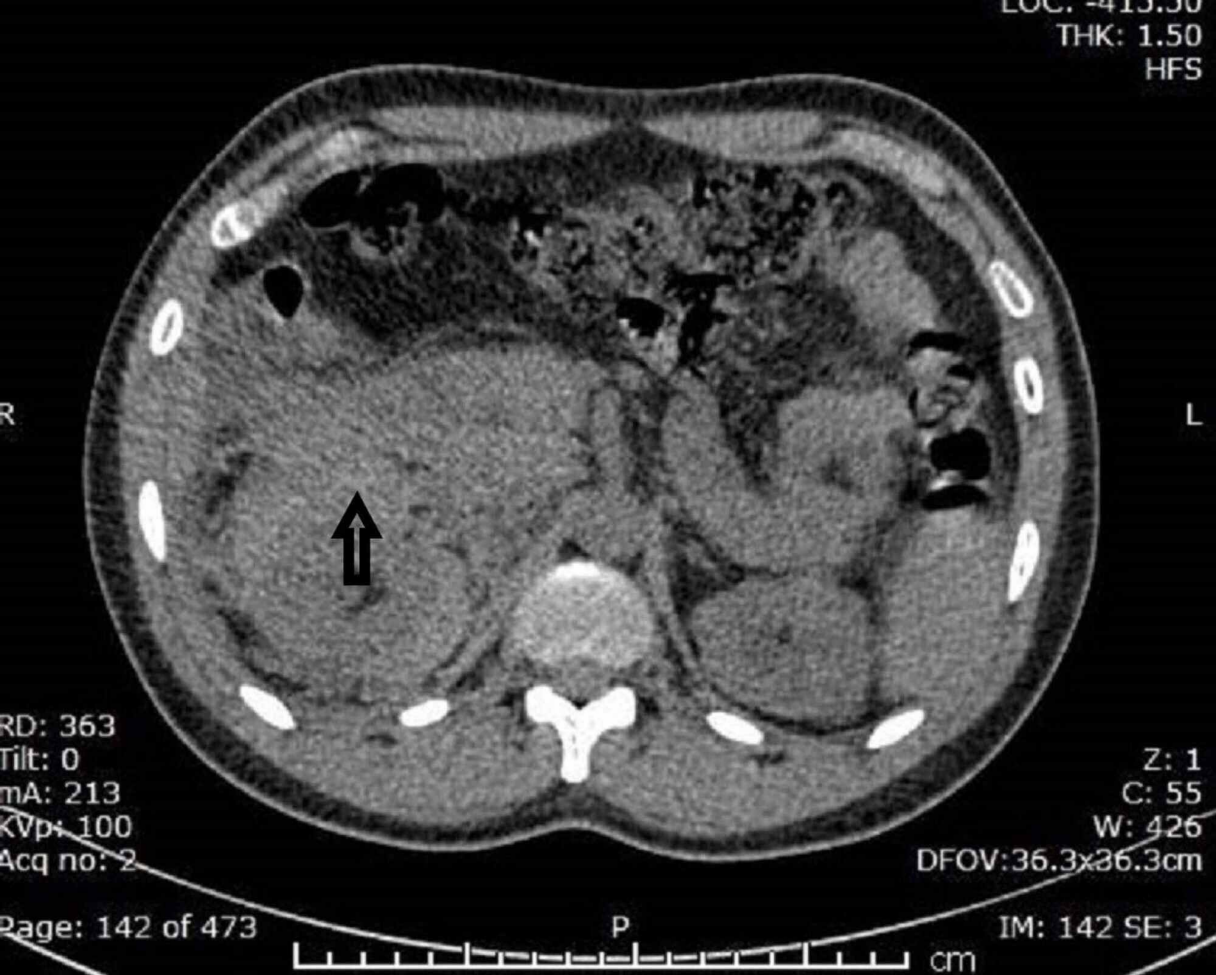
Noncontrast CT-KUB showing enlarged right kidney with perinephric fat stranding and isodense lesion in the perinephric space (arrow), showing a CT density of recent bleed (45-50 HU). CT-KUB, computed tomography-kidney ureter bladder; HU, Hounsfield unit

Given his persistent pain over the flank region, with renal dysfunction, a noncontrast computed tomography-kidney ureter bladder (CT-KUB) was performed, showing enlarged right kidney with perinephric fat stranding and isodense lesion in the perinephric space, showing a CT density of recent bleed [(45-50 Hounsfield unit (HU)] (Figure 1).

**Figure 2:**
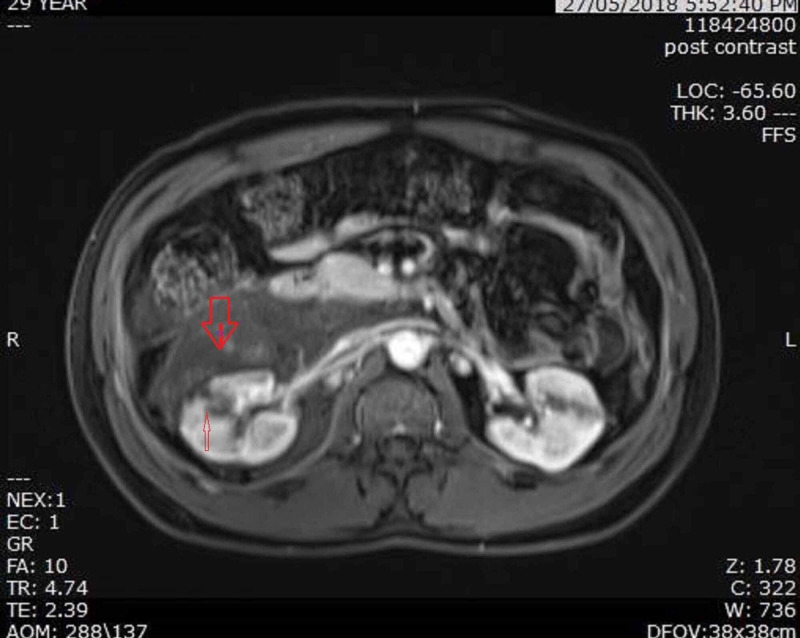
MRI showing large perinephric hematoma (arrow head) and a small hemorrhagic cyst ( arrow) in anterior renal cortex communicating with perinephric hematoma.

The patient was hospitalized for further workup and management and was started on analgesics and antibiotics. After two days of hospitalization, the patient underwent an MRI abdomen with contrast, showing a large perinephric hematoma (arrowhead) and a small hemorrhagic cyst (arrow) in the anterior renal cortex communicating with perinephric hematoma (Figure 2).

He was treated symptomatically. The patient was pain-free and hemodynamically stable. The urology team was consulted, who advised for conservative management and close observation. He did not have a further fall in Hgb or worsening of renal function. Further workup for other causes, like vasculitis, were negative.

**Figure 3:**
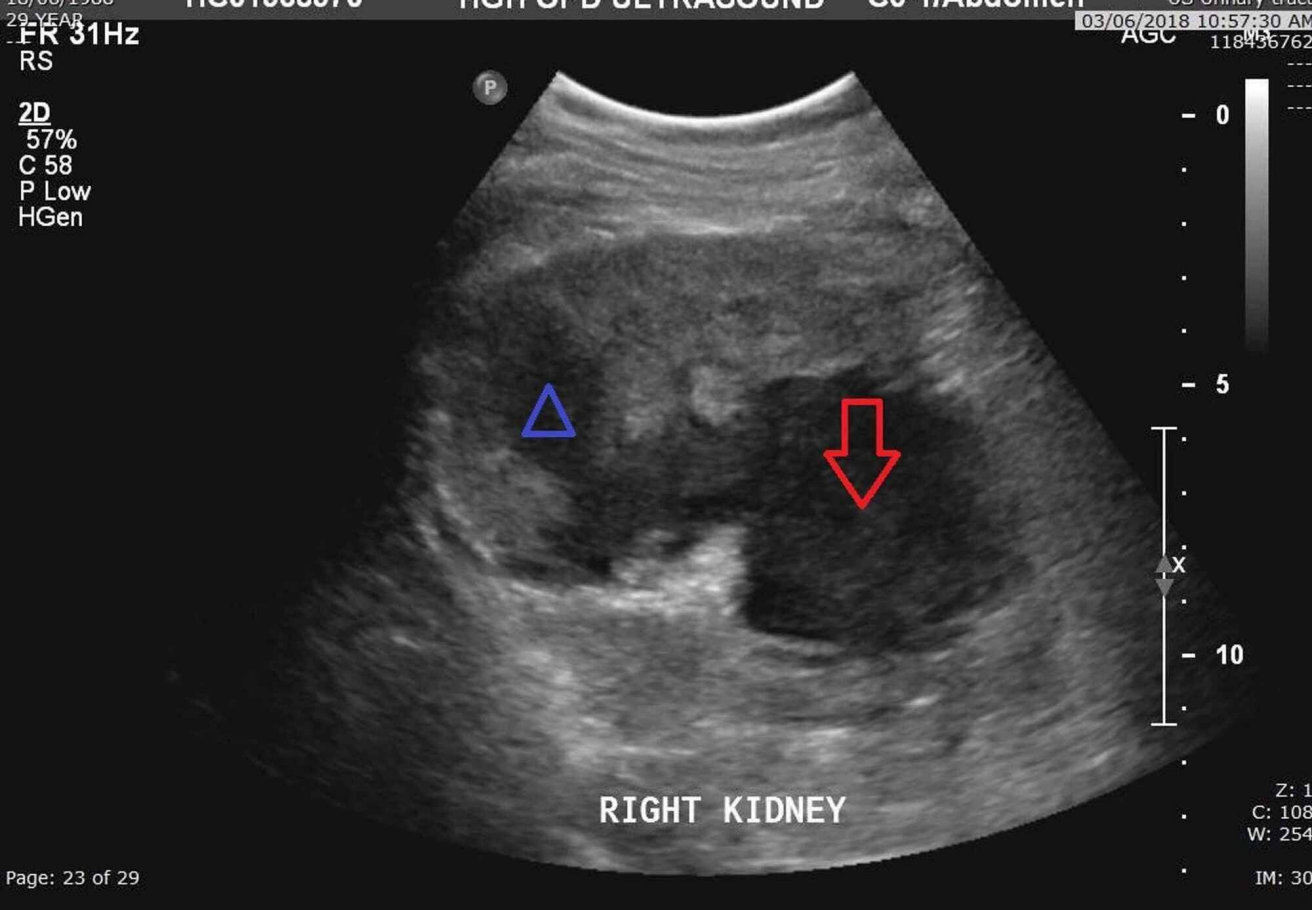
Ultrasound: showing perinephric hematoma (arrowhead) communicating with hemorrhagic cyst (arrow).

On the eighth day of admission, ultrasound imaging of the kidney revealed an estimate of 208 cc of perinephric fluid collection (Figure 3). 

**Figure 4:**
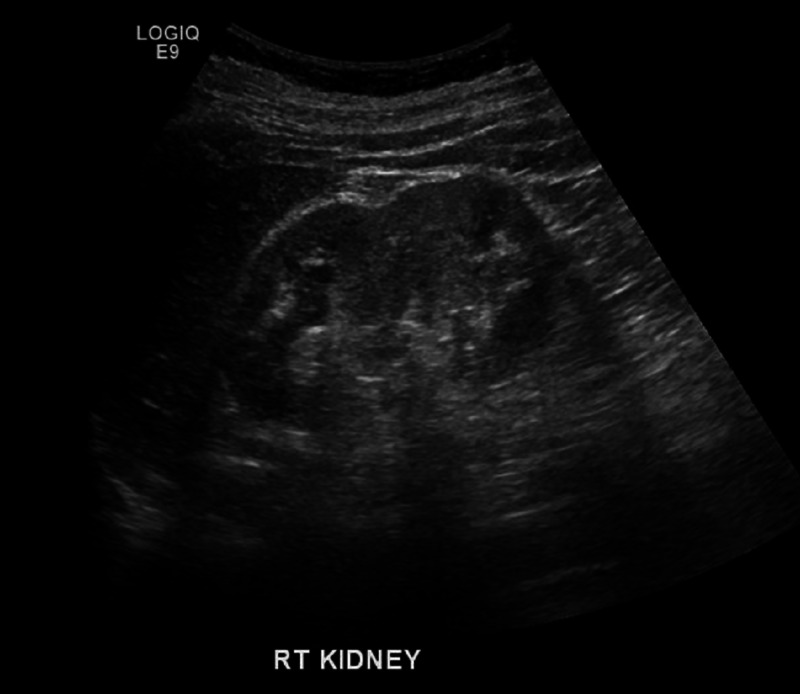
Follow-up ultrasound after three months showing total resolution of perinephric hematoma.

As the patient improved symptomatically, he was discharged with outpatient follow-up. Follow-up ultrasounds done in the first and third weeks after discharge demonstrated regression of hematoma. Follow-up ultrasound done after three months postdischarge demonstrated complete resolution of hematoma, and no evidence of malignancies was detected (Figure 4).

## Discussion

Wunderlich syndrome is a rare clinical syndrome characterized by a sudden onset of spontaneous, nontraumatic hemorrhage into the renal subcapsular and retroperitoneal region [3]. This syndrome was first described by Wunderlich in 1856 [4]. A meta-analysis in 2002 by Zhang et al. described 165 patients of spontaneous nontraumatic hemorrhage over 15 years between 1985 and 1999 [2].

Clinical presentation is often characterized by a constellation of symptoms known as the Lenk’s triad -- acute onset flank pain, palpable flank mass, and hypovolemic shock [4]. However, a study observed that the typical presentation was seen in only 20%-30% of the cases [5]. 

Other presentations include fever, nausea, vomiting, and hematuria. The most common underlying pathology in up to 60% of cases are neoplasms which mostly include renal angiomyolipoma and renal cell carcinoma [3]. These neoplasms arise from the proliferation of epithelioid cells present around the blood vessels. Renal cell carcinoma is the most common malignant neoplasm. Rupture of the renal artery, arteriovenous malformation, nephritis, renal calculi, cystic medial necrosis, segmental arterial mediolysis, polyarteritis nodosa, and cystic rupture are the other common etiologies [2, 4]. 

No underlying etiology could be found in our patient, as seen in a few other reported Wunderlich syndrome cases. In our case, the initial presentation mimicked renal colic. Early diagnosis can be aided by accurate clinical assessment and appropriate imaging. The initial imaging of choice is an ultrasound abdomen, although abdominal CT has a 100% sensitivity [6-8].

The MRI of the abdomen further confirmed our diagnosis. Exploratory laparotomy or nephrectomy is done for most patients. However, in our case, as no underlying malignancy was detected on initial CT, we chose to opt for conservative management with close clinical monitoring and follow-up imaging. This option can also be considered in stable patients with no evidence of malignancy on CT/MRI. This conservative approach can avoid the need for surgery.

## Conclusions

It would be prudent to remember Wunderlich syndrome as a differential diagnosis in patients presenting with unexplained abdominal pain to ED.
